# Quantification of Indocyanine Green Fluorescence Imaging in General, Visceral and Transplant Surgery

**DOI:** 10.3390/jcm12103550

**Published:** 2023-05-18

**Authors:** Lukas Pollmann, Mazen Juratli, Nicola Roushansarai, Andreas Pascher, Jens Peter Hölzen

**Affiliations:** Department for General, Visceral and Transplant Surgery, University Hospital Münster, 48149 Munster, Germany; pollmann.lukas@yahoo.de (L.P.); mazen.juratli@ukmuenster.de (M.J.); nicola.roushansarai@ukmuenster.de (N.R.); andreas.pascher@ukmuenster.de (A.P.)

**Keywords:** indocyanine green (ICG), near-infrared (NIR) imaging, quantitative evaluation, intraoperative imaging, image-guided surgery, fluorescence imaging

## Abstract

Near-infrared (NIR) imaging with indocyanine green (ICG) has proven to be useful in general, visceral, and transplant surgery. However, most studies have performed only qualitative assessments. Therefore, a systematic overview of all studies performing quantitative indocyanine green evaluation in general, visceral, and transplant surgeries should be conducted. Free term and medical subject heading (MeSH) term searches were performed in the Medline and Cochrane databases until October 2022. The main categories of ICG quantification were esophageal surgery (24.6%), reconstructive surgery (24.6%), and colorectal surgery (21.3%). Concordantly, anastomotic leak (41%) was the main endpoint, followed by the assessment of flap perfusion (23%) and the identification of structures and organs (14.8%). Most studies examined open surgery (67.6%) or laparoscopic surgery (23.1%). The analysis was mainly carried out using manufacturer software (44.3%) and open-source software (15.6%). The most frequently analyzed parameter was intensity over time for blood flow assessment, followed by intensity alone or intensity-to-background ratios for structure and organ identification. Intraoperative ICG quantification could become more important with the increasing impact of robotic surgery and machine learning algorithms for image and video analysis.

## 1. Introduction

Pushing the boundaries of intraoperative imaging, ICG is a well-established agent for various surgical fields including general, visceral, and transplant surgery [1]. After intravenous administration, it binds to plasma proteins and is extracted from the liver [2]. Allergic reactions are the most reported but rare side effect, occurring in 0.05–0.07% of all administrations [3]. Many studies on esophageal [4,5] and colorectal surgery [6] recommend the use of ICG imaging to study intestinal perfusion prior to anastomosis. For instance, Zehetner et al., assessed the perfusion of the gastric conduit using ICG and NIR imaging during esophagectomy [5]. When anastomosis was performed in an area of poor perfusion, the incidence of anastomotic leak was significantly higher than that in a well-perfused area.

Furthermore, NIR imaging with ICG in reconstructive surgery was recommended for perfusion assessment of locoregional and free flaps [7].

Similar to the assessment of cutaneous and intestinal blood flow, the blood flow of kidney or liver transplants can be visualized immediately after intravenous administration [8,9] and a correlation with postoperative graft function was shown.

In hepatobiliary surgery, ICG fluorescence imaging has proven to be a sensitive tool for liver segment staining, identification of the biliary system, and liver tumors [10]. For visualization of liver segments, positive staining can be performed intraoperatively by segmental portal vein or artery puncture [11] or negative staining with clamping of the segmental pedicle and intravenous application [12]. Staining of liver segments has been used during liver resection [13,14] as well as for living liver donor transplantation [15]. If administered preoperatively, cholangiography can be performed. Thus, several studies of laparoscopic cholecystectomy indicate the superiority of ICG and NIR imaging for the identification of the biliary tree compared to wide-light visualization alone [12,16]. In liver tumors, biliary excretion is locally disrupted and can be used to visualize the tumor [17]. The authors specify a depth of 8 mm as a limitation of this application. Therefore, NIR imaging should be used in addition to intraoperative ultrasound for tumor visualization [12].

Furthermore, Kim et al., highlighted the role of intraoperative ICG imaging in thyroidectomy and parathyroidectomy to preserve parathyroid function [18], and Gálvez-Pastor et al. [19] developed a scoring system for parathyroid vascularization that was significantly associated with postoperative hypocalcemia.

However, most studies, reviews and meta-analysis have described a qualitative and surgeon-dependent interpretation of the fluorescence signal that is affected by inter-user variability [20,21]. Consequently, the impact of NIR imaging with ICG on intraoperative decision-making and the comparability of existing studies is impaired. Therefore, for the first time, this review aims to summarize the published studies that quantitatively measured intraoperative ICG fluorescence signal. Additionally, an overview of the current ICG analysis techniques is provided, and various indications as well as future directions for ICG imaging are discussed.

## 2. Materials and Methods

**Figure 1:**
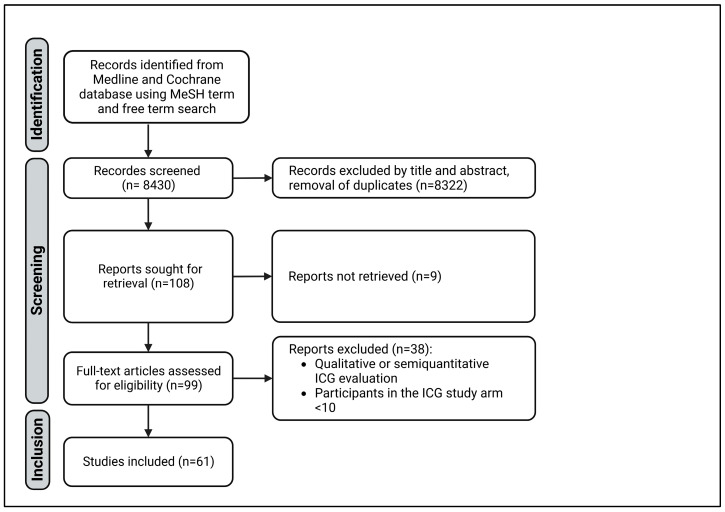
PRISMA diagram of study selection.

A systematic literature search was carried out on studies with intraoperative measurement of ICG fluorescence according to the rules of the preferred reporting items for systematic reviews and meta-analyses (PRISMA) guidelines [22]. The Medline and Cochrane databases were searched by two independent reviewers using the free terms ‘surgery’ and ‘indocyanine green’ and the MeSH terms ‘green, indocyanine’ and ‘evaluation, quantitative’ and ‘surgery’. The results were last updated on 31 October 2022. All clinical studies with quantitative analysis of intraoperative ICG imaging in general, visceral, and transplantation surgeries with more than 10 participants were included. Studies from other surgical disciplines, as well as experimental, animal, and cadaveric studies, were excluded. The study was not included if the number of participants was fewer than 10 or if the abstract contained a purely qualitative ICG assessment. Next, duplicates were removed. Found reports were retrieved. Finally, the articles were then checked for eligibility using a full-text search (Figure 1).

This study was registered with the registration number 412003 at the Prospero registration website. The risk of bias was assessed using the ROBIS tool.

The included studies were compared using predefined criteria. The ICG amount, time of ICG administration, NIR camera system, and quantification software were analyzed in all studies. Subsequently, the type of operation and intraoperatively monitored organs or structures were examined. Next, the studies were categorized into surgical fields. Finally, the measured parameters and endpoints of the studies were summarized.

## 3. Results

### 3.1. Systematic Search

The free term and MeSH term searches identified 8430 studies with 7980 and 450 results, respectively. After the screening, 61 studies were included in this review (Figure 1). All variables are shown in Supplementary Materials Table S1. The risk of bias was considered low. However, using the ROBIS tool, the heterogeneity of the included studies was identified as a weak point of this review, impairing the synthesis of the studies.

### 3.2. Characteristics of Included Studies

**Figure 2:**
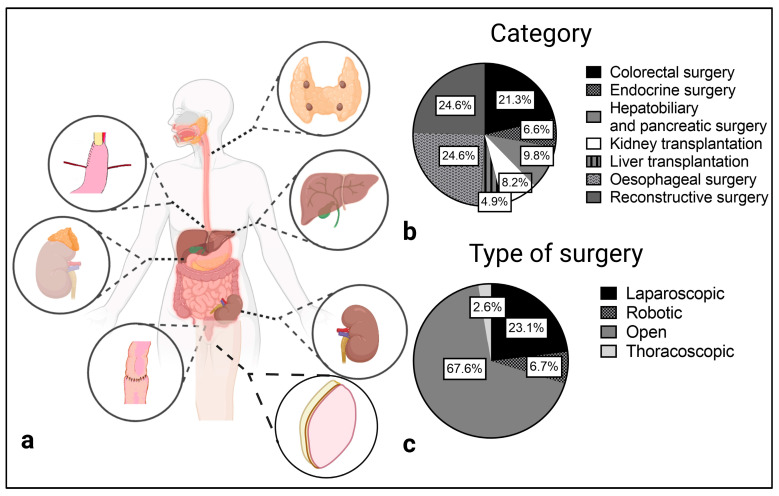
Surgical categories (**a**,**b**) and type of surgery (**c**) of the included studies.

Reconstructive (*n* = 15), esophageal (*n* = 15) and colorectal (*n* = 13) surgery were the main categories in which quantitative analysis was performed. Reconstructive surgery was solely performed as open surgery. In esophageal surgery most studies used an open approach as well (*n* = 10 of 15), while colorectal surgery was mostly performed laparoscopically. Six studies reported intraoperative ICG quantification in hepatobiliary and pancreatic surgery. Except for laparoscopic cholecystectomy, all operations were performed openly. Overall, only one study of fully robotic esophageal resection [23] and one study which utilizes intraoperative ICG imaging in robotic adrenalectomy [24] reported a robotic operation technique (Figure 2).

The two main approaches for ICG quantification were organ and structure identification and quantification of the perfusion as a functional assessment of the tissue or organ of interest. The latter can be done by plotting the intraoperative recorded ICG fluorescence intensity over time. Afterwards the maximum intensity (Fmax), time to reach maximum intensity (Tmax), and halved values with Fmax(1/2) or Tmax(1/2) can be determined. Another frequently mentioned coefficient is the slope of the ICG curve, which is defined as Fmax/Tmax. Furthermore, the ingress as an inflow parameter and the egress describing the outflow of ICG were calculated in several studies with the SPY-Q Software [25,26].

**Figure 3:**
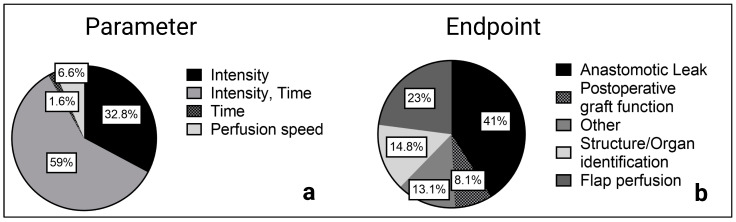
Reported parameters (**a**) and primary endpoints (**b**) of the included studies.

In esophageal surgery, perfusion assessment of the gastric conduit or the jejunal graft has been reported most frequently to predict the occurrence of anastomotic leak (Figure 3). For example, Lin et al. [27] measured the perfusion in a free jejunal graft for esophageal reconstruction and was able to predict a high-risk for anastomotic leak. In their study a Tmax(1/2) value greater than 5.35 s was correlated with the development of anastomotic leak. Recently, de Groot et al. [23] assessed the perfusion of a gastric conduit for full robotic esophageal resection and reconstruction with a gastric pull-up. Although not significant, a higher Tmax value was associated with anastomotic leak. In addition, Ishikawa et al. [28] evaluated ICG fluorescence at the tip and 5 cm from the tip of the gastric conduit and showed a significant correlation between delayed ingress time between the two points and anastomotic leak.

Koyanagi et al. [29] focused on perfusion speed and its relationship with anastomotic leak by measuring the length of the gastric conduit from the pylorus to the tip. Next, a subjective assessment of the time from the start of perfusion at the pylorus to the tip of the gastric conduit was performed, and the speed with length/time was calculated. The speed of the vascular arcade supplying the gastric conduit and the serosal ICG speed of the gastric conduit were compared. A delay in serosal perfusion compared with the vascular arcade was significantly associated with the occurrence of an anastomotic leak [29].

Similar to esophageal surgery, the primary goal of intraoperative ICG quantification in colorectal surgery was the perfusion assessment of the intestinal anastomosis to predict the occurrence of an anastomotic leak. In the study by Wada et al. [30] and Amagai et al. [31], a higher Tmax value was significantly associated with the occurrence of an anastomotic leak, whereas Gomez-Rosado et al. [32] highlighted the slope as a predictive parameter for anastomotic leak. In addition, a delay in the parameter T0, which describes the time from administration to the first detected increase in ICG fluorescence, was indicative of an anastomotic leak in the studies by Hayami et al. and Iwamoto et al. [33,34].

Perfusion assessment of locoregional or free flaps is also crucial for reconstructive surgery. Possible complications of an inadequate blood supply include necrosis of the skin at the margin of the locoregional or free flap, wound infections, prolonged healing, or complete loss of the transplanted graft. The use of intraoperative ICG quantification in breast reconstruction has been reported most frequently (*n* = 11 of 15 studies). For example, Girard et al. [35] described different ingress values in the deep inferior epigastric perforator (DIEP) flap depending on the distance to the perforator. However, no association with clinical complications was found. In head and neck reconstruction using autologous free flaps, Schöpper et al. [36] identified the Fmax/min value, defined as the Fmax divided by the background ratio, as a predictor of flap necrosis.

In kidney transplantation, Gerken et al. [26] demonstrated a significant correlation between a delayed reperfusion ingress of the graft and the development of delayed graft function, as previously suggested [9,37,38,39]. Similar results were reported by Dousse et al. [8] in liver transplantation. In their study, they were able to show a significant correlation between delayed graft reperfusion and the primary nonfunction of the liver transplant.

Structure or organ identification represents the second, central approach of intraoperative ICG quantification. In general, this goal can be achieved by measuring maximum intensity values or calculating maximum intensity-to-background ratios.

For instance, in laparoscopic cholecystectomy, the common biliary duct as an important structure was identified by calculating the intensity of the common biliary duct to liver parenchyma ratio in the studies of Pujol-Cano et al. [40] and Chen et al. [41].

In addition, differentiate liver regions with venous occlusion [42] or ischemia [13] were identified based on intensity-to-background ratios. Furthermore, identification of demarcation and resection lines [13,14] were facilitated during open liver resection by an intensity-to-background measurement. Similar to hepatobiliary surgery, two studies of liver transplantation performed living-donor hepatectomy and described the use of quantitative ICG assessment to identify regions of venous occlusion [43] or the line of demarcation [15].

Furthermore, Shirata et al. [44] described the use of intraoperative ICG quantification in open pancreatic surgery to identify and differentiate pancreatic lesions by calculating a pancreatic lesion to surrounding pancreatic parenchyma ratio. In contrast, the identification of liver tumors as reported by Wakabayashi et al. [10] could not be included in this review, because solely qualitative identification has been reported up to 31 October 2022.

Finally, quantitative ICG imaging and the calculation of intensity-to-background ratios for structure and organ identification has also been described in endocrine surgery. For example, Iritani et al. [45] assessed the perfusion of the parathyroid gland after thyroidectomy. In their study, the maximum intensity of the parathyroid gland was determined before and after the administration of ICG and a ratio was calculated. Concordantly, a lower ratio was associated with the development of a postoperative hypoparathyroidism. For the detection of parathyroid adenomas in hyperparathyroidism, Le Cui et al. [46] administered ICG one hour prior to surgery and were able to identify the adenoma utilizing a modified intensity-to-background ratio. However, most studies identifying the parathyroid gland took advantage of the higher autofluorescence of this organ in NIR imaging [47] without administration of ICG.

Overall, ICG intensity over time with the described parameters was examined most frequently (*n* = 36 of 61 studies), especially in perfusion assessment before anastomosis in esophageal and colorectal surgery as well as for skin and flap assessment in reconstructive surgery. Maximum intensity and intensity-to-background ratios were also frequently reported (*n* = 20 of 61 studies), mainly when a structure or organ was identified, for example, in hepatobiliary or endocrine surgery.

### 3.3. Administered ICG Dosages for Perfusion Assessment and Structure Identifictation

The administered ICG dose was mentioned in 50 of 61 reports, with more studies using an absolute ICG dose (24 studies) than a weight-dependent dose. The greatest lack of information on ICG dosages was found in hepatobiliary and pancreatic surgery (*n* = 2 of 6), followed by reconstructive surgery (*n* = 4 of 15), such as in the study by Mazdeyasna et al. [48].

For a comparison of reported absolute ICG dosages and weight-dependent ICG dosages, the absolute ICG dosages were converted to a relative ICG dose using a body weight of 80 kg. Furthermore, dosages were organized with respect to the application in perfusion assessment with intensity over time curves or organ/structure identification utilizing intensity alone or intensity-to-background ratio.

**Figure 4:**
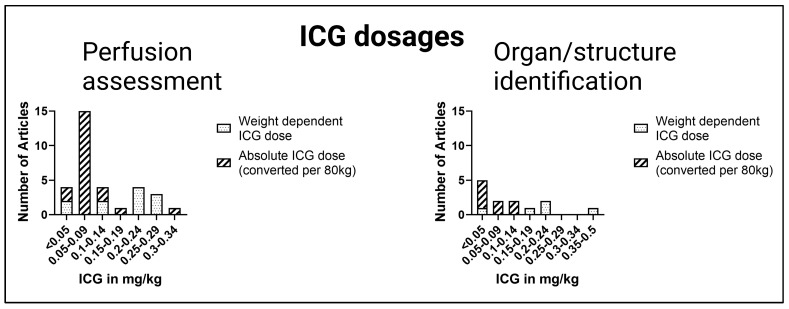
Comparison of ICG dosages. Absolute ICG dosages were converted to weight-dependent ICG dosages (estimated bodyweight: 80 kg).

Figure 4 illustrates the administered ICG doses ranging from <0.05 mg/kg to 0.5 mg/kg in the included articles of perfusion assessment as well as for structure and organ identification. Overall, studies with an absolute ICG dose tended to administer a lower ICG dose than studies with a weight-dependent ICG dose. In summary, mostly lower ICG dosages with <0.05 mg/kg and 0.05–0.09 mg/kg were reported.

For perfusion assessment in esophageal and colorectal surgery, a wide range of ICG amounts from 0.06–0.25 mg/kg have been used. In reconstructive surgery, the administered ICG dosages varied from 0.06–0.13 mg/kg. For imaging of the graft in transplant surgery, ICG dosages of 0.02–0.31 mg/kg were administered. In hepatobiliary surgery, a liver volume-dependent dose was used by Kawaguchi et al. [13,42] instead of a weight-dependent dose. When cholangiography was performed, such as during laparoscopic cholecystectomy, ICG was administered prior to surgery. Additionally, to visualize the parathyroid gland, Le Cui et al. [46] recommended an ICG dose of 0.5 mg/kg one hour before the operation. In contrast, the parathyroid glands were imaged immediately after ICG administration in the study of Noltes et al. [49]; see Table 1.

### 3.4. NIR Camera System and Software for ICG Quantification

**Figure 5:**
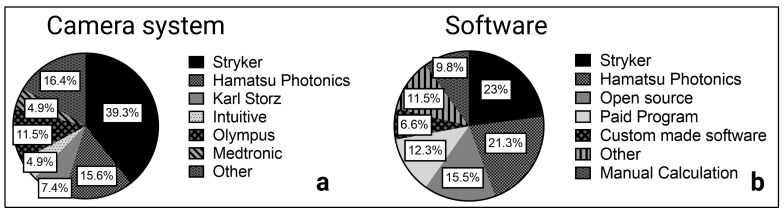
Camera system (**a**) and software (**b**) in ICG quantification.

The included studies showed the use of various camera systems. Stryker (Kalamazoo, MI, USA) was used in 24 studies and developed the SPY Elite system specifically for open surgery [9,26,28,39,49,50,51] and the PINPOINT camera system for minimally invasive surgery [15,33,44,52]. PINPOINT, with the developing manufacturer Novadaq (Seattle, WA, USA), was independent until 2017; therefore, Novadaq instead of Stryker can be found in reports up to 2017. In addition, Karl Storz (Tuttlingen, Germany) and Olympus (Tokyo, Japan) developed NIR camera systems mainly for minimally invasive surgery with IMAGE S1 [34,37,53,54,55] and Viscera Elite II, respectively. The photodynamic eye (PDE) camera system from Hamatsu Photonics (Shizuoka, Japan), the VisionSense (VS) iridium camera from Medtronic (Minneapolis, MN, USA), and the HyperEye Medical System (HEMS) from Mizuho Medical (Tokyo, Japan) are designed for open surgery. Of these, the PDE camera system was most frequently used in open surgery studies (*n* = 9). Robotic surgery was performed using the da Vinci surgical system with a Firefly NIR camera. Other manufacturers of the included NIR camera systems were OptoMedic Technology (Foshan, China), Fluoptics (Grenoble, France), Pulsion Medical Systems (Feldkirchen, Germany), and Beijing Aisery Medical (Bejing, China) (Figure 5).

Intraoperative ICG fluorescence of the recorded videos and selected frames were analyzed using the manufacturer software in 27 studies (Figure 5). The ROI Software of Hamatsu Photonics [56,57] measures the ICG inflow as fluorescence intensity over time in one or more predefined regions of interest (ROI). Using the Spy-Q Software [26,58] by Stryker, the ingress of ICG fluorescence describing the inflow of ICG in the ROI and the egress as outflow were measured, as previously mentioned.

The most frequently used open-source software was Fiji, which allows the manual measurement of pixel intensity values. This function is used to calculate the intensity-to-background ratio of an illuminated structure or organ, such as the thoracic duct [52], parathyroid glands [46], or adrenal tumors [24]. When different frames are examined, intensity over time curves can be created, and the depicted values (Tmax, Fmax, etc.) can be calculated as described by de Groot et al. [23]. Commonly used paid programs with the ability to measure pixel intensities and calculate an intensity-to-background ratio were Adobe Photoshop and Image-Pro Plus. For instance, they have been used to identify the common hepatic duct in contrast to the liver parenchyma [40,41].

Some studies (*n* = 4 out of 46 studies) focused on the development of custom software for ICG fluorescence quantification. In a recent study on anastomotic leakage in colorectal surgery, Park et al. [59] developed a machine learning algorithm based on unsupervised learning with self-organizing map (SOM) clustering of ICG fluorescence over time curves. Unlike supervised learning approaches, learning does not require ground truth from a specialist. With a fixed number of clusters, the learning process is performed on a training data set without human annotation. After clustering, the high-risk clusters for anastomotic leak were identified and an ICG curve classification model was trained depending on the clinical outcome. Once the SOM clustering and ICG curve classification models have been trained, prediction based on the newly recorded ICG fluorescence curves was possible. This machine learning approach achieved a higher F1-Score, which represents an average score of recall and precision for the occurrence of an anastomotic leak versus the time ratio (Tmax(1/2)/Tmax) and slope of the median ICG curve.

## 4. Discussion

There is a large body of literature suggesting that intraoperative NIR imaging with ICG is helpful for various indications in general, visceral and transplant surgery. However, recent findings describe an inter-user variability that limits the subjective assessment of fluorescence imaging, thus impairing intraoperative decision-making [20,21]. In a retrospective randomized study by Larsen et al. [20], intraoperative NIR imaging with ICG during low-anterior rectum resection was recorded and videos were exchanged between the performing surgeons of the participating centers. Subjective assessment showed a wide inter-observer variation unrelated to anastomotic leak, while quantitative assessment using fluorescence over time curves with differences in the slope was significantly associated with the development of anastomotic leak.

Although studies performing quantitative assessment still represent the minority of all reports of NIR imaging with ICG, the existing literature emphasizes development towards objective assessment, quantification, and evaluation. Therefore, for the first time, this systematic review aimed to summarize all studies that performed a quantitative analysis of intraoperative ICG NIR imaging in general, visceral, and transplantation surgery.

After identification, screening, and assessment for eligibility, 61 studies were included in this review. Although the PRISMA guidelines were followed, the reproducibility of results is limited by the steady increase of new articles in the emerging field of ICG imaging in general, visceral, and transplant surgery. Our results were last updated on 31 October 2022.

Starting with the amount of ICG used, we observed a differentiation in a weight-dependent, weight-independent, and organ-specific administration. It must be mentioned that some papers did not specify an ICG dosage. The majority of studies reported an absolute ICG count. Interestingly, these studies tended to use smaller dosages when converted to a mean body weight of 80 kg. As stated above, the mean body weight was based on current epidemiologic data for industrial states, and the authors are aware of this source of error. In addition, hepatobiliary studies by Kawaguchi et al. [13,42] reported a liver volume-dependent ICG dose that was appropriate given the biliary hepatic excretion of ICG. However, this method requires radiological image data or intraoperative volume estimation, which can complicate its clinical implementation. Overall, we recommend that the ICG amount should be reported in the material and methods part and be administered depending on the body weight to ensure comparability.

Regarding the time of administration, NIR imaging was mostly performed intraoperatively immediately after ICG administration to visualize the blood flow in the organ or structure of interest. Only in hepatobiliary surgery, due to the biliary excretion of ICG [10] and one study to identify the parathyroid glands [46], was NIR imaging performed several hours after application.

The camera systems Hamatsu Photonics (Shizuoka, Japan) (PDE), Stryker (Kalamazoo, MI, USA) (SPY Elite), and Medtronic (Minneapolis, MN, USA) (VisionSense VS Iridium) are commonly used in open surgery, while Olympus (Tokyo, Japan) (Viscera Elite II), Karl Storz (Tuttlingen, Germany) (IMAGE S1), and Stryker (PINPOINT) have developed camera systems for minimally invasive surgery. Most of the commercialized systems are in auto-settings, limiting the possibility of comparing intra-patient and inter-patient data. Furthermore, several studies have suggested that fluorescence intensity changes with the distance of the camera from the structure or organ of interest [60,61]. For example, Serra-Aracil et al. [60] showed a significant correlation between the distance from the desired anatomy with the measured fluorescence intensity in a prospective study of colorectal surgery. Therefore, finding the optimal distance remains complex and depends not only on the bioavailability of ICG, but also on technical modalities like the NIR imaging system with the variability of excitation light sources, emission filters, lens optical properties, sensitivity of fluorescence signal detectors, and processing software that make standardization difficult. In future studies, the selected NIR imaging system and recommended distance from the organ of interest might be standardized using ex vivo ICG imaging phantoms or calibration with standardized fluorescent samples similar to white balance in white light laparoscopy.

In future research, robotic approaches could increase [62,63] and the rate of open surgeries will decrease accordingly. Therefore, as the main manufacturer, the intuitive Firefly camera could be more widely used in future research.

In addition to the heterogeneity of the camera systems, heterogeneity in the software used and the reported parameters was also observed. Many studies have performed fluorescence measurements using manufacturer software such as ROI (PDE) and SPY-Q (Stryker). While only ICG inflow parameters were determined by the ROI software, SPY-Q measures the inflow as ingress and the outflow as egress. Parameters determined from intensity over time curves, such as Fmax1/2, Tmax1/2, and ingress, correlated significantly with anastomotic leak. Furthermore, they have a large impact on perfusion assessment in reconstructive surgery as well as for the graft function in kidney and liver transplantation. In summary, inflow parameters seem to be crucial for graft survival as well as for cutaneous and intestinal blood flow. For structure or organ identification, the intensity or the intensity-to-background ratio can be recommended.

In a recent study, Park et al. [59] developed a custom-made machine learning approach that clusters and analyzes intensity over time curves in relation to the risk of anastomotic leak and showed a superiority of his self-organizing map clustering over the most commonly reported calculated parameters. While Park et al., worked with unsupervised, self-organizing map clustering, especially for image and video analysis, there are several machine learning approaches allowing pattern recognition and quantification [64] that have not been tested for intraoperative NIR imaging. Additionally, efforts in various medical fields, particularly radiology [65], indicate the increasing importance of machine learning in medical image analysis. Subsequently, intraoperative ICG imaging could be further developed in future studies with the help of machine learning.

Apart from the included literature, some indications were not reported because of the lack of quantitative assessment. For example, a large body of literature suggests the possibility of ICG-guided lymphadenectomy during gastrectomy [66,67]. The study by Okubo et al. [68] indicates a difference in the intensity-to-background ratio between sentinel lymph nodes and non-sentinel lymph nodes in early gastric cancer and recommended intraoperative, quantitative measurement in future studies. In this study, ICG was applied to the tumor side, and the removed lymph nodes were examined ex vivo.

Furthermore, in a pilot study on cytoreductive surgery, peritoneal carcinosis due to colorectal cancer was observed using NIR after ICG administration [69]. As initial results, the authors showed the benefit of NIR with ICG compared to white light alone in the detection of non-mucinous peritoneal metastases. This resulted in a change in the surgical plan in 30% of the surgeries. To distinguish between fibrosis or other benign lesions and metastases, objective evaluation might be helpful in future studies.

Moreover, tumor visualization in hepatobiliary surgery has been described in several studies, and the optimal timing of administration and dosage of ICG have already been discussed by Wakabayashi et al. [10]. However, NIR imaging of liver tumors has not yet been quantified, and the identification of liver lesions is still performed subjectively by surgeons. This could be due to the different fluorescent types of liver lesions [17,70] complicating a simple intensity or intensity-to-background ratio approach used mainly in structure or organ identification. Fluorescence imaging is also limited due to differences in liver function and ICG extraction capacity, particularly in patients with impaired liver function. Furthermore, regenerate nodes in cirrhotic livers also appeared to be fluorescent in the study by Tanaka et al. [71], which made tumor identification more difficult. However, these problems may be solved by developing sophisticated machine learning and pattern recognition approaches in future studies.

In summary, the results of this review show that quantitative intraoperative ICG assessment is widely used in general, visceral, and transplant surgery and could gain importance with developments in robotic surgery and machine learning. Further indications for quantitative ICG evaluation may arise in future studies. Currently, intraoperative ICG imaging is limited by heterogeneity in reported ICG dosages, different NIR camera systems, and variability in the examined parameters, as well as different software for quantification. Therefore, future research should focus on standardization of ICG dosage, time of administration, imaging modalities, and quantification [49].

## Figures and Tables

**Table 1:** ICG dosages and time of administration.

Method	Surgery	Number of Articles	ICG in mg/kg	Time of Administration
**Perfusion** **Assessment**	Laparoscopic or robotic colon resection	12	0.06–0.25	Intraoperative
	Open colon resection	1	0.06	
	Open esophagectomy with jejunal interposition	2	0.01	Intraoperative
	Minimally invasive or open Mc Keown or Ivor–Lewis esophagectomy	13	0.02–0.3	Intraoperative
	Flap perfusion assessment in head and neck surgery	2	0.06–0.13	Intraoperative
	Flap perfusion assessment in reconstructive breast surgery	9	0.06–0.13	
	Assessment of the graft in kidney transplantation	4	0.02–0.31	Intraoperative
	Assessment of the graft in liver transplantation	1	0.01	Intraoperative
**Structure/** **Organ** **Identification**	Robotic adrenalectomy	1	0.06	Intraoperative
	Open thyroidectomy	2	0.03–0.06	
	Open parathyroidectomy	1	0.5	One hour prior to surgery
	Open liver resection	3	0.03 or liver volume calculated	Intraoperative
	Open pancreatic surgery	1	0.03	
	Laparoscopic cholecystectomy	2	0.003–0.125	Anesthetic induction to 10–12 h prior to imaging
	Living donor hepatectomy in liver transplantation	2	0.02–0.1	Intraoperative

## Data Availability

The authors confirm that the data supporting the findings of this study are available within the article and its Supplementary Materials.

## Supplementary Materials

The following supporting information can be downloaded at: https://www.mdpi.com/article/10.3390/jcm12103550/s1, Table S1: Summary of included studies and parameters [72,73,74,75,76,77,78,79,80,81,82,83,84].
