# Supplementary material for: Quantification of Indocyanine Green Fluorescence Imaging in General, Visceral and Transplant Surgery
Source: J Clin Med. 2023 May 18;12(10):3550. doi: 10.3390/jcm12103550 (PMC10219543; doi:10.3390/jcm12103550)
Supplement: Supplementary file 1 [file jcm-12-03550-s001.zip › jcm-2361860-supplementary.pdf]

Table S1. Summary of included studies and parameters.

Author , Year	Category	Number of patients	Type of surgery	Camera system	Software	Parameter	Endpoints	ICG in mg (absolute)	ICG in mg/kg	Timing
Protyniak, 2015	Colorectal surgery	77	Laparoscopic	Stryker	Stryker	Intensity	Anastomotic leak	-	-	Intraoperative
Wada, 2017	Colorectal surgery	112	Laparoscopic	Hamatsu Photonics	Hamatsu Photonics	Intensity, Time	Anastomotic leak	5	-	Intraoperative
Son, 2019	Colorectal surgery	86	Laparoscopic	Karl Storz	Open source	Intensity, Time	Anastomotic leak	-	0.25	Intraoperative
Van den Bos, 2019	Colorectal surgery	30	Laparoscopic	Karl Storz, Intuitive	Paid Program	Intensity	Anastomotic Leak	-	0.2	Intraoperative
Hayami, 2019	Colorectal surgery	22	Laparoscopic, Robotic	Karl Storz, Intuitive	Hamatsu Photonics	Intensity, Time	Anastomotic Leak	5	-	Intraoperative
Amagai, 2019	Colorectal surgery	69	Laparoscopic	Olympus	Open source	Intensity, Time	Anastomotic Leak	-	0.2	Intraoperative
Iwamoto, 2020	Colorectal surgery	25	Laparoscopic	Stryker	Hamatsu Photonics	Time	Anastomotic Leak	7.5	-	Intraoperative
Park, 2020	Colorectal surgery	65	Laparoscopic	Stryker	Custom made software	Intensity, Time	Anastomotic Leak	-	0.2	Intraoperative
Han, 2022	Colorectal surgery	22	Laparoscopic, robotic	Olympus, Intuitive	Paid Program	Intensity, Time	Anastomotic Leak	7.5	-	Intraoperative
Gomez-Rosado, 2022	Colorectal surgery	70	Laparoscopic	Medtronic	Other (TIVITA)	Intensity, Time	Anastomotic Leak	7.5	-	Intraoperative

Pfahl, 2022	Colorectal surgery	62	Open	Medtronic	Other (TIVITA)	Intensity, Time	Anastomotic Leak	5	-	Intraoperative
Lobbes, 2022	Colorectal surgery	18	Laparoscopic	Olympus	Open source	Intensity, Time	Anastomotic Leak	5	-	Intraoperative
Arpaia, 2022	Colorectal surgery	11	Laparoscopic	Olympus	Custom made software	Intensity, Time	Anastomotic leak	-	-	Intraoperative
Aydin, 2022	Endocrine surgery	50	Robotic	Intuitive	Open source	Intensity	Other	5	-	Intraoperative
Le Cui, 2017	Endocrine surgery	29	Open	Other	Open source	Intensity	Organ identification	-	0.5	One hour prior to surgery
Noltes, 2021	Endocrine surgery	10	Open	Stryker	Open source, Paid Program	Intensity, Time	Organ identification	5	-	Intraoperative
Iritani, 2022	Endocrine surgery	21	Open	Hamatsu Photonics, Olympus	Hamatsu Photonics	Intensity	Organ identification	2.5	-	Intraoperative
Inoue, 2015	Hepatobiliary surgery	24	Open	Mizuho Medical	Paid Program	Intensity	Structure identification	2.5	-	Intraoperative
Kawaguchi, 2017	Hepatobiliary surgery	21	Open	Hamatsu Photonics	Hamatsu Photonics	Intensity	Structure identification	-	-	Intraoperative
Chen, 2021	Hepatobiliary surgery	24	Laparoscopic	OptoMedic Technology	Paid Program	Intensity	Structure identification	10	-	10 to 12 hours prior to surgery

Pujol-Cano, 2022	Hepatobiliary surgery	44	Laparoscopic	Olympus	Paid Program	Intensity	Structure identification	0.25	-	Anesthetic induction
Kawaguchi, 2013	Hepatobiliary surgery	63	Open	Hamatsu Photonics	Hamatsu Photonics	Intensity	Structure identification	-	-	Intraoperative
Shirata, 2018	Pancreatic surgery	23	Open	Stryker	Hamatsu Photonics	Intensity	Other	2.5	-	Intraoperative
Rother, 2017	Kidney transplantation	57	Open	Stryker	Stryker	Intensity, Time	Postoperative Graft function	-	0.25	Intraoperative
Rother, 2019	Kidney transplantation	77	Open	Stryker	Stryker	Intensity, Time	Postoperative Graft function	-	0.2	Intraoperative
Ellebrecht, 2020	Kidney transplantation	36	Open	Pulsion Medical Systems	Other	Intensity, Time	Postoperative Graft function	25	-	Intraoperative
Gerken, 2022	Kidney transplantation	128	Open	Stryker	Stryker	Intensity, Time	Postoperative Graft function	-	0.02	Intraoperative
Ietto, 2021	Kidney transplantation	37	Open	Karl Storz	Open source	Intensity	Postoperative Graft function	-	0.15	Intraoperative
Hashimoto, 2008	Liver transplantation	21	Open	Other	Manual Calculation	Intensity, Time	Structure identification	-	0.1	Intraoperative
Dousse, 2020	Liver transplantation	76	Open	Other	Manual Calculation	Intensity, Time	Other	-	0.01	Intraoperative

Kim, 2021	Liver transplantation	46	Open	Stryker	Paid Program	Intensity	Other	-	0.025	Intraoperative
Kamiya, 2015	Esophageal surgery	26	Open	Hamatsu Photonics	Hamatsu Photonics	Intensity, Time	Other	-	-	Intraoperative
Yukaya, 2015	Esophageal surgery	27	Open	Mizuho Medical	Other	Intensity, Time	Anastomotic Leak	-	0.1	Intraoperative
Koyanagi, 2016	Esophageal surgery	40	Open	Hamatsu Photonics	Manual Calculation	Perfusion speed	Anastomotic Leak	2	-	Intraoperative
Ishige, 2019	Esophageal surgery	20	Open	Olympus	Hamatsu Photonics	Intensity, Time	Anastomotic Leak	1.25	-	Intraoperative
Sugimura, 2019	Esophageal surgery	20	Open	Hamatsu Photonics	Hamatsu Photonics	Intensity, Time	Other	12.5	-	Intraoperative
Nerup, 2020	Esophageal surgery	10	Open, Laparoscopic, Thoracoscopic, Robotic	Karl Storz	Custom made software	Intensity, Time	Anastomotic Leak	-	0.25	Intraoperative
Talavera-Urquijo, 2020	Esophageal surgery	100	Laparoscopic, Thoracoscopic	Olympus	Manual Calculation	Perfusion speed	Anastomotic Leak	-	0.3	Intraoperative
Koyanagi, 2021	Esophageal surgery	109	Open	Hamatsu Photonics	Manual Calculation	Perfusion speed	Anastomotic Leak	1.25	-	Intraoperative
Ishikawa, 2022	Esophageal surgery	304	Laparoscopic, Thoracoscopic, Robotic	Stryker	Stryker	Intensity, Time	Anastomotic Leak	5	-	Intraoperative
Barnes, 2022	Esophageal surgery	20	Laparoscopic, Thoracoscopic	Stryker	Open source	Intensity	Other	5	-	Intraoperative

Hennig, 2022	Esophageal surgery	13	Open	Medtronic	Other (TIVITA)	Intensity	Anastomotic Leak	-	-	Intraoperative
Lin, 2022	Esophageal surgery	84	Open	OptoMedic Technology	Hamatsu Photonics	Intensity, Time	Anastomotic Leak	0.75	-	Intraoperative
Von Kroge, 2022	Esophageal surgery	20	Open	Stryker	Custom made software	Intensity, Time	Anastomotic Leak	-	-	Intraoperative
Ninomiya, 2022	Esophageal surgery	140	Open	Hamatsu Photonics	Manual Calculatio n	Perfusion speed	Anastomotic Leak	1.4	-	Intraoperative
de Groot, 2022	Esophageal surgery	63	Robotic	Intuitive	Open source	Intensity, Time	Anastomotic Leak	7.5	-	Intraoperative
Mastronardi, 2022	Reconstructive surgery	34	Open	Stryker	Open source	Intensity, Time	Flap perfusion	-	0,2	Intraoperative
Mazdeyasna, 2022	Reconstructive surgery	11	Open	Stryker	Paid Program	Intensity	Flap perfusion	-	-	Intraoperative
Van den Hoven, 2021	Reconstructive surgery	13	Open	Other	Other	Intensity, Time	Flap perfusion	7,5	-	Intraoperative
Schöpper, 2022	Reconstructive surgery	67	Open	Other	Other (Flow 800)	Intensity, Time	Flap perfusion	10	-	Intraoperative
Varela, 2020	Reconstructive surgery	51	Open	Hamatsu Photonics	Hamatsu Photonics	Intensity	Flap perfusion	-	0,2	Intraoperative
Girard, 2019	Reconstructive surgery	40	Open	Stryker	Stryker	Intensity, Time	Flap perfusion	5	-	Intraoperative
Adelwahab, 2019	Reconstructive surgery	71	Open	Stryker	Stryker	Intensity, Time	Flap perfusion	5	-	Intraoperative
Yang, 2018	Reconstructive surgery	10	Open	Stryker	Stryker	Intensity, Time	Flap perfusion	-	-	Intraoperative

Guo, 2018	Reconstructive surgery	30	Open	Stryker	Stryker	Intensity, Time	Flap perfusion	10	-	Intraoperative
Wang, 2018	Reconstructive surgery	17	Open	Stryker	Stryker	Intensity, Time	Flap perfusion	7,5	-	Intraoperative
Gorai, 2017	Reconstructive surgery	81	Open	Stryker	Hamatsu Photonics	Intensity, Time	Flap perfusion	2	-	Intraoperative
Surowitz, 2015	Reconstructive surgery	10	Open	Stryker	Stryker	Intensity	Flap perfusion	-	-	Intraoperative
Cho, 2015	Reconstructive surgery	10	Open	Stryker	Stryker	Intensity	Other	-	-	Intraoperative
Munabi, 2014	Reconstructive surgery	42	Open	Stryker	Stryker	Intensity	Flap perfusion	10	-	Intraoperative
Losken, 2012	Reconstructive surgery	77	Open	Stryker	Stryker	Intensity, Time	Flap perfusion	5	-	Intraoperative
